# Impact of SARS-CoV-2 infection in patients with hereditary hemorrhagic telangiectasia: epidemiological and clinical data from the comprehensive Italian retrospective multicenter study

**DOI:** 10.1007/s11739-023-03287-8

**Published:** 2023-05-04

**Authors:** Patrizia Suppressa, Eugenia Maiorano, Eleonora Gaetani, Elina Matti, Gennaro Mariano Lenato, Ilaria Serio, Maristella Salvatora Masala, Giulio Cesare Passali, Maria Aguglia, Claudia Crocione, Pietro Luigi Lopalco, Francesca Caneschi, Valeria Musella, Annalisa De Silvestri, Giulia Gambini, Giuseppe Spinozzi, Carlo Sabbà, Fabio Pagella

**Affiliations:** 1 “Frugoni” Internal Medicine and Geriatrics Unit, Centro Sovraziendale per le Malattie Rare, DIM-Interdisciplinary Department of Medicine, HHT Interdepartmental Center, VascERN HHT Reference Center, Policlinico Hospital, University of Bari, Bari, Italy; 2 Department of Otorhinolaryngology, Fondazione IRCCS Policlinico San Matteo, Pavia, Italy; 3 Internal Medicine and Gastroenterology Unit, Department of Medical and Surgical Sciences, Multidisciplinary Gemelli Group for HHT, Fondazione Policlinico Universitario Agostino Gemelli IRCCS, Università Cattolica del Sacro Cuore School of Medicine, Rome, Italy; 4 Division of Internal Medicine, Department of Medical and Surgical Sciences, IRCCS Azienda Ospedaliero-Universitaria di Bologna, University of Bologna, Bologna, Italy; 5 Clinica Medica Unit, Sassari University Hospital, Sassari, Italy; 6 Division of Otorhinolaryngology, Multidisciplinary Gemelli Group for HHT, Fondazione Policlinico Universitario Agostino Gemelli IRCCS, Università Cattolica del Sacro Cuore School of Medicine, Rome, Italy; 7 Clinical Pathology Unit, Vito Fazzi Hospital, Lecce, Italy; 8 HHT Onlus Patient Association, Rome, Italy; 9 Department of Biological and Environmental Science and Technologies, University of Salento, Lecce, Italy; 10 University of Pavia, Pavia, Italy; 11 Clinical Epidemiology and Biometry Unit, Scientific Direction, Fondazione IRCCS Policlinico San Matteo, Pavia, Italy

**Keywords:** Hereditary hemorrhagic telangiectasia, Rare diseases, COVID-19, SARS-CoV-2 infection, Arteriovenous malformation

## Abstract

Rare Disease patients manifested high concern regarding the possible increased risk of severe outcomes and worsening of disease-specific clinical manifestation due to the impact of COVID-19. Our aim was to assess the prevalence, outcomes, and impact of COVID-19 in patients with a rare disease such as Hereditary Hemorrhagic Telangiectasia (HHT) in Italian population. A nationwide, multicentric, cross-sectional observational study was conducted on patients with HHT from five Italian HHT centers by online survey. The association between COVID-19-related signs and symptoms and nosebleeds worsening, the impact of personal protective equipment on nosebleeds pattern, and the relationship between the presence of visceral AVMs and severe outcomes were analyzed. Out of 605 total survey responses and eligible for analysis, 107 cases of COVID-19 were reported. A mild-course COVID-19 disease, not requiring hospitalization, was observed in 90.7% of patients, while the remaining eight cases needed hospitalization, two of them requiring intensive-care access. No fatal outcome was recorded and 79.3% of patients reported a complete recovery. No difference in infection risk and outcome between HHT patients and general population was evidenced. No significative interference of COVID-19 on HHT-related bleeding was found. The majority of patients received COVID-19 vaccination, with relevant impact on symptoms and need for hospitalization in case of infection. COVID-19 in HHT patients had an infection profile similar to the general population. COVID-19 course and outcome were independent from any specific HHT-related clinical features. Moreover, COVID-19 and anti-SARS-CoV-2 measures did not seem to affect significantly HHT-related bleeding profile.

**Supplementary Information:**

The online version contains supplementary material available at 10.1007/s11739-023-03287-8.

## Introduction

Hereditary Hemorrhagic Telangiectasia (HHT) is a rare disease (RD) characterized by epistaxis, mucocutaneous telangiectases, and visceral arteriovenous malformations (AVMs) with a prevalence of 1:6,000 [1, 2]. It is transmitted as an autosomal dominant trait and displays two major genetic subtypes, known as HHT1 and HHT2, due to disease-causing mutations in the *ENG* gene (endoglin) and in the *ACVRL1* gene (Activin A Receptor type II-Like Kinase 1), respectively [3]. *ENG* and *ACVRL1* mutations are responsible for 85% of HHT cases, with mutations in *SMAD4* accounting for another 2% of cases and the remainder cases of as-yet unknown genetic origin [3–5]. The mutations lead to an angiogenesis imbalance ultimately resulting in multiple vascular shunts, consisting in abnormal connections between the artery and vein lacking intervening capillary bed, known as telangiectases, when affecting skin and/or mucosa, or as AVMs when affecting the vascular network of internal organs [6, 7]. Epistaxis, the leading sign of HHT and the most frequent clinical manifestation, occurs earlier in HHT1 and shows an increasing trend in HHT2 subjects [1, 2].

Since this is a RD, with important manifestations mainly in the rhinological area, as well as in some internal organs, it is of particular interest to understand whether any specific HHT-related manifestation implies increased risk to infection of Severe Acute Respiratory Syndrome Coronavirus 2 (SARS-CoV-2), or correlates with more severe SARS-CoV-2 infection-associated adverse outcome. COVID-19, caused by SARS-CoV-2, has represented a worldwide health emergency that has seen Italy as the first European epicenter of the pandemic spread [8]. The heterogeneity of the clinical manifestations of COVID-19 is well known in the literature, ranging from asymptomatic or paucisymptomatic presentations to cases requiring hospitalization and often invasive ventilatory support [9]. Advanced age and the presence of comorbidities such as hypertension, obesity, and diabetes mellitus are known risk factors for the development of severe COVID-19, but little is known about the role of rare conditions such as HHT [10, 11].

Multisystem involvement and complications related to the presence of visceral AVMs, such as chronic anemia, dyspnea, portal and pulmonary hypertension, brain abscesses, high-flow heart failure, may represent potential risk elements for severe forms of the disease. Last but not least, the role of nosebleeds, which make patients with HHT more prone to nasal manipulations, should be focused, as it might represent an increased potential risk of exposure to SARS-CoV-2 [12, 13].

Very few data are currently available concerning the prevalence and impact of SARS-CoV-2 infection in the HHT population, or the influence of COVID-19 on the systemic and rhinological clinical manifestations of HHT [14–16]. Nationwide epidemiological investigations were limited to an inquiry carried on in the Spanish population, assessing pneumonia-associated hospitalization [15], and to a multicenter survey in the Italian population performed by our national network, characterizing infection risk, phenotypic spectrum and need for intensive care [14], with a third study in the Spanish population based on a single-center recruitment [16].

However, such studies were only focused on the first pandemic wave, characterized by a limited infection spreading, partly mitigated through early lockdown measure implementation, whereas no data are reported in the literature upon restarting SARS-CoV-2 virus circulation across the subsequent pandemic waves. Furthermore, no study has tried to elucidate the preventative effect of anti-SARS-CoV-2 vaccination in the subsequent waves. The need to further collect additional data in the characterization of the SARS-CoV-2-infection impact on HHT subjects prompted us to expand our previous survey to a longer period, spanning the entire pandemic emergency. Hence, the objective of the present study was to investigate the role of HHT disease as a risk factor for SARS-CoV-2 infection susceptibility, the contribution of HHT to severity of COVID-19 clinical course, and the relationship between HHT-related features, SARS-CoV-2 infection, attitudes toward wearing personal protective equipment, in the Italian HHT population.

## Methods

### Study design

The study is a nationwide questionnaire-based survey, with a multicenter retrospective cross-sectional design, addressed to the whole Italian HHT population. Patients were recruited from December 1, 2021 to March 1, 2022 through an anonymous web-based data collection survey that was disseminated via email in a dedicated newsletter by HHT Onlus (Italian Association of HHT Patients) and by the five Italian HHT Centers of expertise involved in the study (Bari University “Policlinico” Hospital VascERN Center, Bologna IRCCS University Hospital, Pavia Fondazione IRCCS “Policlinico San Matteo” University Hospital, Rome Fondazione IRCCS “Policlinico Gemelli” Catholic University Hospital, Sassari University Hospital). An e-mail reminder was sent after 15 days to the same contacts. HHT Onlus also disseminated the survey via website (www.hhtonlus.org) and through all social media platforms. The survey was addressed to patients with a genetically or clinically confirmed diagnosis of HHT [3, 17–19]. No selection criteria were applied in the patient enrolment phase, as the survey addressees were recruited independent of the presence/absence of any visceral involvement and/or complications, mutated gene, or severity of HHT-related manifestations. Prior to survey compiling, an information form explaining the aim of the study and the anonymous mode of data collection was presented to the patients. The study was approved by the Ethics Committee of all the five participating Institutions.

### Survey

The survey was a semi-structured interview composed of two sections. The first section included information concerning COVID-19 vaccination, eventual SARS-Cov-2 infection and re-infection, such as sign and symptom onset throughout the entire pandemic period and type (fever, coughing, headache, dyspnea, muscular pain, sense of tiredness/fatigue, diarrhea, sore throat, nasal obstruction, rhinorrhea, conjunctivitis, and gustative/olfactory sensory impairment), diagnostic procedures, clinical course and outcomes (resolution, need for hospitalization and ventilatory support, and death). Specific questions distinguished ascertained COVID-19 cases, based on laboratory-testing-based confirmation of diagnosis (namely, positive results on at least one of the following laboratory investigations: SARS-Cov-2-specific Polymerase Chain Reaction (PCR)-based molecular assay, SARS-Cov-2 antigen test on Nasopharyngeal/Oropharyngeal Swab [14, 20], or anti-SARS-Cov-2 Antibody serological test) [21], from suspected cases, characterized by the subjective reporting of COVID-19-suggestive symptoms lacking molecular/serological confirmation. Subjects reported COVID19-suggestive symptomatology, without laboratory-test confirmation, were excluded from data analysis due to lack of data completeness.

Besides questions regarding general COVID-19 signs and symptoms, the survey collected information concerning specific HHT-typical features during the SARS-CoV-2 infection, such as bleeding profile change and sense of dryness/discomfort of the nasal and oral mucosa, serum iron, serum ferritin and hemoglobin level. Moreover, detailed information regarding the use of personal protective equipment/individual protection devices (PPE/DPI) and potential role in modifying bleeding profile was collected.

The second section collected general data and baseline clinical information, regarding the region of residency throughout the study period, the site of HHT-related AVM involvement (pulmonary AVMs—PAVMs; brain AVMs—BAVMs; hepatic vascular malformations—HVMs; gastrointestinal AVMs—GIAVMs) and the presence of common HHT-unrelated comorbidities. The questionnaire is reported as Supplementary File 1.

### Primary and secondary outcome measures

The survey included questions regarding data encompassing the whole pandemic period, ranging from the pandemic outbreak until the end of the pandemic emergency and the diffusion of anti-SARS-CoV-2 vaccination, collecting data related to a period spanning from November 2019 to February 2022. Estimates of cumulative prevalence rate of SARS-CoV-2 infection were obtained for both whole Italian population and the single Italian Region population. Evaluation of severity of COVID-19 clinical picture was performed by frequency of single COVID-19 manifestations and the presence of severe COVID-19 outcomes (need for hospitalization, ventilatory support or death).

Furthermore, we studied the association between the presence of nasal obstruction, rhinorrhea, cough, pharyngodynia, alterations in smell/taste and variation in nosebleeds and/or bleeding from the oral cavity, the association between the attitude toward wearing personal protective equipment (type and time of use) and variation in nosebleeds and/or bleeding from the oral cavity, the association between the presence of HHT-related visceral AVMs and severe COVID-19 outcomes.

### Patients involvement

Patients advocates from HHT Onlus were involved in the design and conduct of this research project. Priorities of the research, choice of primary and secondary outcome measures, and methods of recruitment were informed by discussions with patients delegates of HHT Onlus that took part in all study development meetings. As results emerged they were reviewed with the patient advocates collecting their perspectives and feedback to ensure that we presented the findings in the most effective way to the general population. This method allowed us to collect valuable contributions from patient advocates during the entire study process while the investigators retained the rigors of scientific work. Guarantee of dissemination of findings to the general population will be ensured also through collaboration with HHT Onlus advocacy activities.

### Statistical analysis and epidemiological comparison

Quantitative variables are described as means and standard deviation; categorical variables are reported as count and percentage. Comparisons between categorical variables are made with chi square test.

For sake of epidemiological comparison, a control group was set up based on administrative data on Italian general population, daily reported and updated by Italian Istituto Superiore di Sanità (ISS) throughout pandemic period, aimed to monitoring epidemic spreading (https://www.epicentro.iss.it/coronavirus/sars-cov-2-dashboard). Observed SarS-Cov-2 cases and hospitalized cases in Italian and Region-specific general population were retrieved on March 8, 2022, when available, at the end of study period. Reference Italian and regional population data were retrieved from ISTAT website (https://www.istat.it/it/popolazione-e-famiglie?dati). For both HHT study group and control general population, SarS-Cov-2 infection prevalence rate and COVID-19 hospitalization rate are reported with 95% confidence interval (95% CI), according to binomial distribution.

## Results

### Baseline clinical and epidemiological features

A total number of 1,418 web-based questionnaire recipients were recruited in the study. Analysis of obtained results showed that a total of 605 patients answered the survey and were eligible for inclusion in data analysis (mean age of 54 ± 17 years; range 6–86 years, female gender ratio = 342/605, 56.5%). Age profile and geographical distribution of the HHT population answering the survey is shown in Table 1.

**Table 1:** Anagraphic profile of the HHT population answering the survey

	*n*/tot (%)
Age class	
0–29	58/593 (9.8%)
30–39	55/593 (9.3%)
40–49	104/593 (17.5%)
50–59	171/593 (28.8%)
60–69	133/593 (22.4%)
≥ 70	72/593 (12.1%)
Residency region	
Abruzzo	11/603 (1.8%)
Aosta valley	0
Apulia	38/603 (6.3%)
Basilicata	0
Calabria	10/603 (1.7%)
Campania	34/603 (5.6%)
Emilia Romagna	50/603 (8.3%)
Friuli Venezia Giulia	13/603 (2.2%)
Latium	75/603 (12.4%)
Liguria	17/603 (2.8%)
Lombardy	121/603 (20.1%)
Marches	37/603 (6.1%)
Molise	10/603 (1.7%)
Piedmont	30/603 (5%)
Sardinia	32/603 (5.3%)
Sicily	39/603 (6.5%)
Tuscany	28/603 (4.6%)
Trentino Alto Adige	18/603 (3%)
Umbria	5/603 (0.8%)
Veneto	35/603 (5.8%)

Clinical and genetic features are reported in Table 2. Epistaxis was the most common sign, with a prevalence of 96.0%. HVMs were reported by 41.7% of patients, followed by PAVMs (35.6%) and GIAVMs (19.7%). A mutation in a known HHT-causing gene was identified in 260 patients, with the highest rate shown by mutations in *ACVRL1* (65.4%), while mutations affecting *ENG* and *SMAD4* were reported in 33.5% and 1.2%, respectively.

**Table 2:** HHT- and non-HHT-related clinical features of the HHT population answering the survey

	*n*/tot (%)
HHT-related clinical manifestations	
Epistaxis	580/604 (96.0%)
HAVMs	228/547 (41.7%)
GIAVMs	110/558 (19.7%)
BAVMs	63/544 (11.6%)
PAVMs	205/576 (35.6%)
Treated PAVMs	92/203 (45.3%)
Untreated PAVMs	111/203 (54. 7%)
Mutated gene	
ENG	87/260 (33.5%)
ALK1/ACVRL1	170/260 (65.4%)
SMAD4	3/260 (1.2%)
Non-HHT related comorbidities	
Diabetes	25/605 (4.1%)
Hypertension	133/605 (21.9%)
Asthma	45/605 (7.4%)
Stroke	21/605 (3.5%)
Obesity	29/605 (4.5%)
Chronic kidney disease	11/605 (1.8%)
COPD	21/605 (3.5%)
Pulmonary hypertension	17/605 (2.8%)
Oncologic disease	22/605 (3.6%)
Allergy	148/605 (24.5%)
Smoking	93/605 (15.3%)

Remote medical history of the HHT population answering the survey, including HHT- and non-HHT-related clinical features

*HHT* Hereditary hemorrhagic telangiectasia, *HAVMs* hepatic arteriovenous malformations, *GIAVMs* gastrointestinal arteriovenous malformations, *BAVMs* brain arteriovenous malformations, *PAVMs* pulmonary arteriovenous malformations, *COPD* chronic obstructive pulmonary disease

### COVID-19 cases

Among the surveys collected, 107 cases of COVID-19 were reported, with a prevalence of 17.5, with a mean age of 46 ± 16 years, ranging from 6 to 75. Confirmation of diagnosis was carried out by molecular/antigen assay on nasopharyngeal swab in 25.5% of cases, on oropharyngeal swab in 62.3% of cases, and serological test on peripheral blood in 15.1% of cases. An additional six subjects, reporting COVID19-suggestive symptomatology but lacking laboratory-test confirmation, were considered to be uneligible due to incomplete data, thus being ruled out from the study. Of the 107 recorded HHT patients with suspected and confirmed COVID-19, 13 subjects reported occurrence of SARS-CoV-2 reinfection.

Clinical spectrum of COVID-19 was variable, ranging from asymptomatic patients to severe manifestations, requiring hospitalization. Table 3 shows in details the clinical features during the SARS-CoV-2 infection in the 107 HHT patients with confirmed COVID-19.

**Table 3:** Clinical symptomatology reported by the 107 HHT patients with confirmed COVID-19

	*N* (%)
Clinical symptomatology	
Fever	59/107 (55.1%)
Coughing	47/107 (43.9%)
Headache	29/107 (27.1%)
Dyspnea	20/107 (18.7%)
Muscular pain	46/107 (43%)
Sense of tiredness/fatigue	57/107 (53.3%)
Diarrhoea	15/107 (14%)
Sore throat	26/107 (24.3%)
Nasal obstruction	35/107 (32.7%)
Rhinorrhea	22/107 (20.6%)
Conjunctivitis	10/107 (9.4%)
Gustative/olfactory sensory impairment	40/107 (37.4%)
None	8/107 (7.5%)

Regarding the clinical course of SARS-CoV-2 infection, 97 patients (90.7%) had a mild-course domiciliary-management-based COVID-19, while in eight cases (7.6%) patients needed hospitalization, two of them requiring also requiring intensive-care-unit admission, with the two remaining cases not reporting this data. Oxygen supplementation through continuous positive airway pressure (CPAP) therapy was reported by five patients, while none of the patients required invasive ventilation, tracheotomy, or extracorporeal membrane oxygenation (ECMO). Analyzing COVID-19 outcome at the time of survey compilation, 79.3% of patients reported a complete recovery, 16% suffered from persistent post-acute symptomatology despite SARS-CoV-2 laboratory-based-testing negativization and 4.7% reported complete COVID-19 clinical resolution despite prolonged positivity at laboratory virological testing. No fatal outcome was recorded.

### COVID‐19 and HHT‐related manifestations

As far as the potential effect of HHT-related manifestations and COVID-19 are concerned, nor the presence of PAVMs (*p* = 0.19), HVMs (*p* = 0.33) and/or GIAVMs (*p* = 0.22) were statistically associated with the clinical course and the outcome of SARS-CoV-2 infection. None of the patients requiring intensive care monitoring had hemoglobin levels ≤ 7 g/dl, serum iron ≤ 49 mcg/dl and serum ferritin ≤ 20 ng/dl. Of the 107 patients with ascertained COVID-19, 50 had a known genetic mutation in *ENG* (18/50), *ACVRL1* (31/50) or *SMAD4* (1/50). Among the 50 COVID-19 patients with identified disease-causing mutation, hospitalization was needed in four cases, represented by one *ENG*-mutated patient and three *ACVRL1*-mutated patients, two of the latter requiring intensive care monitoring.

### COVID‐19 and HHT‐related bleeding

Throughout Sars-Cov-2 infection phase, epistaxis severity showed no variation in 67.3% (72/107) of patients, while the remaining patients reported a variation in nosebleed severity pattern, manifesting as an increase in 16.8% (18/107) and as a decrease in 15.9% (17/107) of respondents, respectively.

Overall, 60.7% of patients revealed no significant nasal discomfort, whereas dryness (20.6%), wetness (13.1%) and nasal pain (5.6%) were infrequent. Bleeding from oral telangiectases showed no variation in 94.9% (94/99) of patients, while 4.1% (4/99) and 1% (1/99) of patients reported an increase or decrease, respectively, in bleeding severity. The profile of gastrointestinal bleeding was unmodified in the 95.7% (88/92) of cases, improved in 1.1% (1/92) and worsened in 3.2% (3/92). The analysis between COVID-19 signs and symptoms and HHT bleeding profile showed no correlation, except for the presence of perceptive gustative/olfactory impairment and an increase in epistaxis frequency and intensity (*p* = 0.012) and rhinorrhea and the increase in bleeding from oral telangiectases (*p* = 0.031).

### Effect of personal protective equipment/individual protection devices

All the respondents stated that they used to wear facemasks during the study period (homemade, surgical, FFP2, FFP3). The most commonly PPE/DPI was surgical mask (52.6%), followed by FFP2 (45.3%). Nor the type of PPE/DPI nor the wearing time (less than 1 h/day, from 1 to 4 h/day; more than 4 h/day) affected the bleeding profile significantly. Hand hygiene measures (gloves, alcoholic solutions, frequent hand washing) were adopted by 105 patients, with only 1 suffering from cutaneous telangiectasia bleeding as complication.

### COVID-19 prevalence

Data on COVID-19-related prevalence rate and hospitalization rate in HHT group were compared with a control group represented by general Italian population at the time of the survey, as shown in Table 4. Prevalence estimates were compared in whole Italian general population, as well as in the three Regions with higher respondents’ frequency in our survey. The raw cumulative prevalence rate of ascertained COVID-19 cases in HHT was 107/605 (0.1769, 95% CI 0.1486–0.2093), showing no statistically significant difference with respect to Italian general population (0.2084, 95% CI 0.2083–0.2085), as mirrored by 95% CI overlap. Similarly, as also shown in Table 4, Region-specific comparison evidenced no difference between raw cumulative COVID-19 prevalence rate in patients living in Lombardy (0.2314, 95% CI 0.1596–0.3168), Piedmont (0.3000, 95% CI 0.1473–0.4939), and Emilia-Romagna (0.3000, 95% CI 0.1473–0.4939), with the respective regional general population. Likewise, no statistically significant difference was found in COVID-19-related hospitalization risk between HHT (8/107 hospitalized cases; hospitalization rate: 0.0748, 95% CI 0.0384–0.1407) and Italian general population (hospitalization rate: 0.0444, 95% CI 0.0443–0.0446).

**Table 4:** Comparison between raw prevalence rates and hospitalization rate of COVID-19 among HHT patients and general population

	HHT		General population	
Prevalence rate (HHT vs. general population)	Observed cumulative COVID-19 cases	Raw cumulative prevalence (95% CI)	Observed cumulative COVID-19 cases	Raw cumulative prevalence (95%CI)
Lombardy	Observed cases: 28 Population: 121	0.2314 (0.1596–0.3168)	Observed cases: 2,366,486 Population: 9,943,004	0.2380 (0.2377–0.2383)
Piedmont	Observed cases: 9 Population: 30	0.3000 (0.1473–0.4939)	Observed cases: 979,505 Population: 4,256,350	0.2289 (0.2285–0.2293)
Emilia-Romagna	Observed cases: 13 Population: 50	0.2600 (0.1463–0.4034)	Observed cases: 1,200,531 Population: 4,425,366	0.2713 (0.2709–0.2717)
Italy	Observed cases: 107 Population: 605	0.1769 (0.1486–0.2093)	Observed cases: 12,428,641 Population: 59,630,133	0.2084 (0.2083–0.2085)
Hospitalization rate (HHT vs. general population)	Observed cumulative COVID-19 hospitalized cases	Raw cumulative hospitalization rate (95% CI)	Observed cumulative COVID-19 hospitalized cases	Raw cumulative hospitalization rate (95% CI)
Italy	Obs. hospitalized cases: 8 Total obs. COVID-19 cases: 107	0.0748 (0.0384–0.1407)	Obs. hospitalized cases: 552,218 Total obs. COVID-19 cases 12,428,641	0.0444 (0.0443–0.0446)

### Effect of COVID-19 vaccination

A total of 564 (92.5%) patients received COVID-19 vaccination and the majority of them (59%) completed the 3-dose vaccination course at the time of the survey compilation (36% received two doses and 5% only the first dose). Of the 107 COVID-19 HHT patients, a considerable proportion (61.3%) of cases was not vaccinated against SARS-CoV-2 infection at the time of COVID-19. Although statistical analysis showed no difference in the number of COVID-19 symptoms in vaccinated vs. unvaccinated patients, the unvaccinated patients represented the majority (80%) of COVID-19-related dyspnea. Likewise, seven of the eight patients requiring hospitalization were unvaccinated.

## Discussion

A number of studies reported increased risk of COVID-19-related averse poor outcomes, such as complications and death, in RD patients, likely due to the typical frailty condition of these patients [22, 23]. An increased risk of COVID-19-related mortality during the first pandemic wave was reported in the UK [24] and in Hong Kong [25], compared to general population. However, these results are mainly based on neurology and neurodevelopmental rare disorders, with very little data available in rare circulatory system disease, such as HHT [26]. HHT is a rare vascular heritable disorder, characterized by mucous and visceral AVMs. In light of the typical multisystemic involvement and the visceral AVM-related complications, such as chronic anemia, dyspnea, portal and pulmonary hypertension, brain abscess, high-flow heart failure, a potentially augmented risk of HHT disease toward SARS-CoV-2-infection susceptibility and/or severe forms of COVID-19 has been inferred [12]. Moreover, the presence of HHT-related recurrent and violent epistaxis, leading to repeated nasal manipulations and frequent access to hospital setting was thought to represent a possible increased risk of SARS-CoV-2 exposure [12, 20, 27]. Conversely, two studies carried on in Spain observed a surprisingly mild degree of manifestations in HHT patients. Riera-Mestre et al. reported only one case out of 1177 HHT patients from Spain admitted for COVID-19 pneumonia [15], while Marcos et al. reported a lower hospitalization rate than general population (2%) and no case of intensive care need [16]. In addition, one of these study also reported a seemingly higher rate of SARS-CoV-2 infection prevalence in HHT compared to general Spanish population [16], which was rather attributed by authors to an augmented testing rate in HHT patients, due to their awareness that their condition might imply a worst outcome. To date, both the risk profiles to develop SARS-Cov-2-infection and to suffer from severe COVID-19 clinical trajectory and outcome in patients with HHT are still uncertain.

In the present work, we reported the results of a nationwide questionnaire-based survey to assess the prevalence of SARS-CoV-2 infection and COVID-19 course and outcome in HHT Italian population throughout the whole pandemic crisis, from the outbreak to the end of the lockdown and the diffusion of anti-SARS-CoV-2 vaccination. Our study showed that the prevalence of COVID-19 in HHT population seemed to parallel the epidemic profile observed in the general population, with overlapping prevalence rates between HHT and general population observed at both national and Region-specific level, thus suggesting that potential SARS-CoV-2 infection susceptibility and/or exposure, such as frequent nose manipulation and immunological impairment, do not seem to confer increased SARS-Cov-2-infection risk in HHT patients.

Regarding the investigation of poor outcome risk, our data highlight a quite benign course with only the 7% needing hospitalization and 2% requiring intensive-care-unit admission in HHT patients. These results are aligned with our preliminary report, centered on the first wave of pandemic spreading [14], and seem to provide further evidence that no significant difference is found on hospitalization rates and severe outcomes in HHT patients when compared to general population, despite the potential coexistence of chronic conditions due to HHT-related multisystemic involvement. Although two previous studies in Spanish population suggested a reduced severity of COVID-19 in HHT patients, the hypotheses advocated to explain this apparent weaker COVID-19 severity still lack confirmation as they are based on the HHT-damaged endothelial function and/or HHT-altered angiogenesis, which would impair the SARS-CoV-2 infection-driven macrophage inflammatory response, thus potentially protecting HHT patients from developing the cytokine storm [15, 16]. Rather, our nationwide survey does not seem to reveal an either increased or reduced severity of COVID-19 in HHT, since the hospitalization rate and the intensive-care-unit treatment rate shows clear statistical overlap with the Italian reference general population. The main strength of our study is that we analyzed, through a nationwide multicentric approach the impact of COVID-19 in HHT patients, collecting several clinical data from 605 patients, which represents an extremely high number given that this is a RD. As noted above, and in our first preliminary survey [14], estimates on hospitalization rates and outcome risks may suffer from significant recruitment bias, whereas such bias can be minimized, although not completely ruled out, by a nationwide multicenter design.

Since saturation of hospital resources represented a major issue during pandemic emergency [28], employment of priority scores in terms of access to hospital care might involve reduced access to life-saving health care in patients with RD such as HHT, a disease characterized by complication risks and reduced lifespan [29–34]. Our results imply relevant clinical implications, as the awareness that HHT is not associated to increased hospitalization risk or mortality in COVID-19 is expected to help appropriate clinical decision-making, in that a lower priority to access to COVID-19 hospital care is not justified for these patients.

As far as the potential effect of HHT systemic manifestation on COVID-19, there was no significant association between the presence of visceral AVMs or chronic iron-deficiency anemia markers and SARS-CoV-2 infection-related clinical course and outcome. When we analyzed the possible impact of COVID-19 on the HHT-related systemic and rhinological clinical manifestations, we found no significant interference between SARS-CoV-2 infection and HHT-related bleeding. The majority of patients reported no variation in epistaxis severity or nasal discomfort during COVID-19, nor modification in gastrointestinal or oral mucosal bleeding profile. The evidence of a significant association between gustative/olfactory impairment and epistaxis severity and between rhinorrhea and bleeding from oral telangiectases is difficult to interpret from a clinical point of view and needs further investigations.

Finally, we investigated the effect of PPE/DPI on skin and mucosal bleeding and the impact of anti-SARS-CoV-2 vaccination campaign. Since many HHT patients were concerned about the possible increase in epistaxis severity due to PPE/DPI wearing, it was fundamental to discover that nor the type of PPE/DPI nor the wearing time affected the bleeding profile significantly. Noteworthy, almost all the survey respondents received COVID-19 vaccination, with the silver lining of a reduced rate of SARS-CoV-2 infection and a benign course with uneventful domiciliary management even in case of infection. This data confirmed the crucial role of the vaccination campaign in order to prevent severe COVID-19 clinical manifestation and to reduce hospitalization.

There are few limitations of our study. First, the retrospective design may not allow to address all possible clinically relevant data and secondly the survey-based inquiry might lead to a recall bias. Consequently, we cannot completely rule out that poor outcome risk might actually be increased in some HHT patients’ subgroups, affected by particular complications, such as PAVM-related dyspnea, brain abscess, or very severe chronic iron-deficient anemia [35], an issue needing further investigations in future studies.

In conclusion, COVID-19 in HHT patients had similar infection profile to general population. COVID-19 and anti-SARS-CoV-2 measures seemed not to interfere with HHT-bleeding profile, nor HHT clinical manifestation affected COVID-19 course and outcome.

## Supplementary Information

Below is the link to the electronic supplementary material.

Supplementary file1 (DOCX 33 KB)

## Data Availability

The data that support the findings of this study are not publicly available, according to decision of the local Ethical Review Board standard operating procedures for cohort studies, which only allow publication of clinical data in aggregate form, to avoid publication of sensitive information that could compromise questionnaire respondents’ privacy.

## Abbreviations

COVID-19: Coronavirus disease 2019
SarS-CoV-2: Severe acute respiratory syndrome coronavirus 2
RD: Rare disease
HHT: Hereditary hemorrhagic telangiectasia
AVM: Arterio-venous malformation
PPE/DPI: Personal protective equipment/individual protection devices
PCR: Polymerase chain reaction
BAVM: Brain arterio-venous malformation
HVM: Hepatic vascular malformation
PAVM: Pulmonary arterio-venous malformation
GIAVM: Gastrointestinal arterio-venous malformation
*ENG*: Endoglin
*ACVRL1*: Activin A receptor type II-Like Kinase 1
CPAP: Continuous positive airway pressure
ECMO: Extracorporeal membrane oxygenation
COPD: Chronic obstructive pulmonary disease
