# Supplementary material for: Impact of SARS-CoV-2 infection in patients with hereditary hemorrhagic telangiectasia: epidemiological and clinical data from the comprehensive Italian retrospective multicenter study
Source: Intern Emerg Med. 2023 May 4;18(4):1109–18. doi: 10.1007/s11739-023-03287-8 (PMC10157588; doi:10.1007/s11739-023-03287-8)
Supplement: Supplementary file 1 — Supplementary file1 (DOCX 33 KB) [file 11739_2023_3287_MOESM1_ESM.docx]

**PART I: COVID 19**

**1) Please help us understand who you are:**

1. I am an HHT patient
2. I am the relative of an HHT patient

**2) Regarding Covid 19:**

1. I had COVID-19
2. I suspect that I had il COVID-19
3. I did not have COVID-19
4. I did not have COVID-19, but I had a tight contact with subjects with ascertained diagnosis of COVID-19 (es. relative, job colleague, family’s doctor, etc)

**3)** **Can you please specify when (MM/YY) you had the first COVID 19 related symptom?**

**4)** **Which specific symptom did you have?**

1. Fever
2. Coughing
3. Headache
4. Dyspnea
5. Muscular pain
6. Sense of tiredness/Fatigue
7. Diarrhoea
8. Sore throat
9. Nasal obstruction
10. Rhinorrhea
11. Conjunctivitis
12. Gustative/olfactory sensory impairment
13. None

**5) Which kind of diagnostic ascertainment did you perform?**

1. Serological Test with blood draw
2. Nasopharyngeal/Nasal Swab
3. Oropharyngeal Swab

**5) Can you please specify when (MM/YY) you had the first Swab Test?**

**…………………………………………**

**5.1)** How many Swab Tests did you perform? …

**5.2)** How many of these Swab Tests gave positive results? …

**5.3)** Did you perform other diagnostic investigations?

1. Lung Echography
2. Lung CT
3. Thoracic X-Ray
4. None

**6) Please describe the course of SARS-CoV-2 infection:**

1. I had a domiciliary management of Covid-19 with no symptoms
2. I had a domiciliary management of Covid-19 with symptoms
3. I was hospitalized
4. I was hospitalized in intensive care unit

**7) In case you were hospitalized, which kind of ventilatory support did you need?:**

1. Oxygen-therapy
2. CPAP
3. Intubation with mechanical ventilation
4. Tracheotomy
5. ECMO (ExtraCorporeal Oxygenation)
6. None

**8) Did you receive psychologic support during hospitalization and care?**

1. Yes
2. No

**9) At moment, how would you define your COVID19-related health status?**

1. I had a 100% recovery
2. I still have some symptoms
3. I am symptom-free but I still have positive swab-testing
4. I recovered but with complications
5. The patient that I am responding about is deceased during SARS-CoV-2 infection

**10) How would you define your nosebleeds during COVID 19?**

1. No change
2. More frequent and intense
3. Less frequent and intense

**11) Please define your sensation about your nose during COVID 19**

1. Very dry
2. Very sweat
3. Painful
4. Unchanged compared to usual

**12) Regarding the gastrointestinal bleeding during COVID 19, did you notice:**

1. Increase of bleeding
2. Reduction of bleeding
3. No change

**13) Regarding the lesions of tongue and oral cavity mucosa, during COVID 19, did you notice:**

1. Increase of bleeding from mouth
2. Reduction of bleeding from mouth
3. No change

**14) Blood values ​​and therapies in the moment you have developed or think you have developed COVID-19**

14.1) My Haemoglobin values were:

1. greater than 14 g/dl
2. greater than 12 g/dl
3. greater than 10 g/dl
4. greater than 8 g/dl
5. greater than 7 g/dl
6. lower than 7 g/dl
7. I don’t know

14.2) My sideremia values were:

A) greater than 53 mcg/dl

B) greater than 49 mcg/dl

C) greater than 45 mcg/dl

D) lower than 45 mcg/dl

E) I don’t know

14.3) My ferritinemia values were:

1. between 20-200 ng/mL
2. lower than 20 ng/mL
3. I don’t know

14.4) I was taking oral iron therapy

14.5) I was being adminsitered intravenous iron therapy

14.6) I was being administered blood transfusions

**15) In the moment you had COVID-19 were you taking any of these drugs?**

1. Cortisone
2. Non-Steroidal Anti-Inflammatory Drugs - NSAIDs
3. Bevacizumab
4. Thalidomide
5. Anticoagulants
6. Anti-hypertensive
7. Anti-hypertensive belonging to ACE-inhibitors category
8. Immunosuppressors
9. Chemotherapy drugs

**16) Which individual protection devices have you worn most?**

1. Home-made facemask
2. Surgical facemask
3. FFP2 facemask
4. FFP3 facemask
5. I have worn no individual protection devices

**17) On average, how long a day have you employed facemask?**

1. < 1 hour/day (e.g. groceries, family issues, etc)
2. between 1 and 4 hours/day
3. > 4 hours/day

**18) Did facemask wearing bring about any changes in your symptomatology?**

**YES NO**

**18.1)** If **YES** can you please specify among the following?:

1. Onset of nosebleeds
2. Worsening of nosebleeds
3. Improvement of nosebleeds
4. Onset of oral bleeding
5. Worsening of oral bleeding
6. Improvement of oral bleeding

**19) Did you carry out individual protection to hands?**

1. gloves
2. alcohol solutions
3. other solutions for hand hygiene
4. frequent hand washing

**20) Did they bring about any complications? YES NO**

**20.1)** If **YES** can you please specify among the following?:

1. Onset of hand telangectasia bleeding
2. Worsening of hand telangectasia bleeding

**21) Did you received anti COVID-19 vaccination? YES NO**

**21.1)** If **YES** Which specific vaccine was administered?:

1. Moderna - Spikevax
2. Astrazeneca – Vaxzevria
3. Johnson & Johnson – Janssen
4. Pfizer - Comirnaty

**21.2)** If **YES** How many vaccine doses were administered?:

1. One
2. Two
3. Three

**22) In case you received anti COVID-19 vaccination, can you specify when (MM/YY) you received the first dose?**

**…………………………………………**

**23) If you are relative of an HHT patient, did your relative with HHT received anti COVID-19 vaccination? YES NO**

**PART II: GENERAL INFORMATION**

**24) General Information**

- Gender  **M F**
- Weight…………….Kg
- Age………… yrs
- Region of residency: ……………...
- Region of staying in the moment of COVID-19: ……………….
- Year of HHT diagnosis: …………
- Reference Center which ascertained diagnosis of HHT: ………………..
- Reference Center of follow-up for HHT: …………………...
- Mutation known: **YES NO**

**24.1**) If **YES** can you please specify the mutation?:

1. HHT1 (ENG - Endoglin)
2. HHT2 (ACVRL1 - ALK1)
3. SMAD4

**PART III: GENERAL HEALTH STATUS AND HHT-RELATED MANIFESTATIONS**

**25) Which of the following HHT manifestations do you have?**

**A B C**

1. Epistaxis a) **YES** b) **NO**
2. Cerebral AVM a) **YES** b) **NO** c) **I DON’T KNOW**
3. Hepatic AVM a) **YES** b) **NO** c) **I DON’T KNOW**
4. Gastrointestinali AVM a) **YES** b) **NO** c) **I DON’T KNOW**
5. Pulmonary AVM a) **YES** b) **NO** c) **I DON’T KNOW**

**25.1)** If **YES** which characteristics do your Pulmonary AVMs have?

1. PAVM waiting for treatment
2. PAVM not needing treatment
3. PAVM already subjected to treatment

**26) Which of the following clinical conditions do you have?**

1. Diabetes
2. Hypertension
3. Asthma
4. Stroke
5. Obesity
6. Chronic kidney disease
7. COPD
8. Pulmonary hypertension
9. Oncologic disease
10. Allergy

**27) Do you smoke?**

1. Yes
2. No
